# Insight into the Structure, Functions, and Dynamics of the *Leptospira* Outer Membrane Proteins with the Pathogenicity

**DOI:** 10.3390/membranes12030300

**Published:** 2022-03-07

**Authors:** Shen-Hsing Hsu, Chih-Wei Yang

**Affiliations:** Kidney Research Center, Department of Nephrology, Chang Gung Memorial Hospital, College of Medicine, Chang Gung University, 5 Fu-Shing St., Taoyuan 33333, Taiwan; d938208@gmail.com

**Keywords:** *Leptospira*, outer membrane lipoprotein, peptidoglycan, Toll-like receptor

## Abstract

Leptospirosis is a widespread zoonosis that frequently occurs in tropical and subtropical countries. *Leptospira* enters the host through wounds or mucous membranes and spreads to the whole body through the blood, causing systemic infection. Kidneys are the preferential site where *Leptospira* accumulates, especially in the renal interstitium and renal tubule epithelial cells. Clinical symptoms in humans include high fever, jaundice, renal failure, and severe multiple-organ failure (Weil’s syndrome). Surface-exposed antigens are located at the outermost layer of *Leptospira* and these potential virulence factors are likely involved in primary host-pathogen interactions, adhesion, and/or invasion. Using the knockout/knockdown techniques to the evaluation of pathogenicity in the virulence factor are the most direct and effective methods and many virulence factors are evaluated including lipopolysaccharides (LPS), *Leptospira* lipoprotein 32 (LipL32), *Leptospira* ompA domain protein 22 (Loa22), LipL41, LipL71, *Leptospira* immunoglobulin-like repeat A (LigA), LigB, and LipL21. In this review, we will discuss the structure, functions, and dynamics of these virulence factors and the roles of these virulence factors in *Leptospira* pathogenicity. In addition, a protein family with special Leucine-rich repeat (LRR) will also be discussed for their vital role in *Leptospira* pathogenicity. Finally, these surface-exposed antigens are discussed in the application of the diagnosis target for leptospirosis and compared with the serum microscope agglutination test (MAT), the gold standard for leptospirosis.

## 1. Introduction

Leptospirosis is a common zoonotic disease transmitted by animals, and the importance of international emerging and re-emerging of infectious diseases as a consequence of global warming and humid environment, particularly common after flooding occurs [1,2,3]. The outbreaks of leptospirosis are accompanied by flooding and heavy rainfall and leptospirosis is considered to be an important disaster-related infectious disease [4]. Leptospirosis caused by pathogenic spirochetes is one of the most neglected zoonotic diseases in tropical and subtropical areas globally [5]. In developed countries, leptospirosis is often associated with travel and adventure. In a United Kingdom study, it was pointed out that nearly half of the confirmed cases of leptospirosis had a history of travel to tropical regions [6]. *Leptospira* infects almost all mammals and rodents that act as a major carrier of *Leptospira*. *Leptospira* are highly motile bacteria that invade the human blood circulation from skin abrasions or mucous membranes, allowing for their rapid dissemination and subsequent colonization of the liver, lungs, and kidneys [5,7]. Approximately 1 million confirmed leptospirosis and approximately 59,000 deaths were found each year [8]. Patients may be asymptomatic or present with a mild headache, muscle pain, and fever to severe pulmonary hemorrhage or meningitis. About 10% of diagnosed patients will develop jaundice, acute kidney injury (AKI), or renal failure, also known as Weil’s disease [9]. Weil’s disease occurs only in severe leptospirosis, manifesting as AKI or renal failure with hepatomegaly and liver function damage [2]. Leptospirosis induced AKI is often characterized by hypokalemia and leptospirosis-related AKI has several interrelated factors characterized by rhabdomyolysis, hyperbilirubinemia, hypovolemia, and direct nephrotoxic effects of *Leptospira* [10]. Diagnosis of leptospirosis is amenable, but it is often too late for critically ill patients, and early penicillin treatment is effective and may dramatically save patients from multiple organ failure [11]. The serum microscope agglutination test (MAT) is the gold standard for leptospirosis, however, MAT has many limitations in clinical applications, and requires the support of high-standard laboratories to accurately test. Besides MAT, many other diagnosis methods have been developed for leptospirosis tests and these methods are discussed in the following paper. The case fatality rate of these patients with severe leptospirosis is 5–20%. Most cases of leptospirosis are mild and resolve with autoimmunity. Early use of antibiotics can further prevent leptospirosis from progressing to severe disease. Therefore, antibiotics can be started once leptospirosis is suspected after a history and examination. Antibiotics such as doxycycline, azithromycin, ampicillin, and amoxicillin are used for the treatment of mild leptospirosis. For severe leptospirosis, intravenous (IV) penicillin is recommended. In addition, amoxicillin, ampicillin, and ceftriaxone can also be used in severe cases. In the process of infection with *Leptospira*, epithelial cells and the immune system trigger an inflammatory response, especially through the production of cytokines. This process is essential for the early elimination of pathogens. However, the overreaction of the immune system leads to uncontrolled cytokine production and a cytokine storm, which may lead to sepsis and related multiple organ failure [12]. According to previous reports, many cytokines are produced after infection with *Leptospira*. Among them, IL-1b, IL-2, IL-4, IL-6, IL-8, IL-10, and TNF-α, which were induced by *Leptospira* virulence factors, were found to be a positive relationship with the severity of the disease of *Leptospira* [13]. For example, Toll-like receptors (TLRs) target the pattern associated molecules (PAM) of *Leptospira*, such as lipoprotein, lipopolysaccharides (LPS), peptidoglycan (PGN), and lipoteichoic acid (LTA), etc. [13,14]. Besides these cell wall and cell membrane components, many virulence factors from the pathogenic Leptospira were identified and the structures and functions of this virulence were well studied in several reviews [15,16,17]. In addition, nucleotide-binding oligomerization domain-containing protein 1 and 2 (NOD1 and NOD2) in the innate immune system are also very important pattern recognition receptors. NOD1 and NOD2 are important for controlling invasion of the extracellular bacteria. In *Leptospira*, NOD1 and NOD2 mainly participate in the identification of PGN of *Leptospira*. Furthermore, to prevent leptospirosis, many related infrastructure and policies need to be further improved, such as improving housing, infrastructure, and sanitation standards, which can reduce the incidence of leptospirosis. Regular rodent control work and flood control projects are also key projects for prevention. In most cases, leptospirosis infection can be prevented by the proper use of personal protective equipment (PPE) by persons at high risk of occupational exposure [18]. In this review, we will focus on the *Leptospira* virulence factors that participated in recognizing of the innate immunity components and inducing the inflammation cytokines expression. Particularly, the regulation and the structure dynamic of *Leptospira* virulence factors to activate the innate immunity components.

## 2. *Leptospira* Membrane Structure and the Virulence Factors

*Leptospira* is a bacterium with a double membrane. Located in the middle of the double membrane is the cell wall of the bacteria. It is mainly composed of PGN and some PGN binding proteins [19,20]. The outermost layer is composed of LPS rich in phospholipids and contains many lipoproteins. LPS is a component of lipid and saccharide on the surface of *Leptospira* species. The *Leptospira* outer membrane contains antigenic components including lipoproteins, lipopolysaccharide (LPS), and peptidoglycans (PGN) [7]. Additionally, *Leptospira* LPS has a low endotoxin activity and this perhaps is a function of the unique structure of *Leptospiral* lipid A [21]. An endotoxic lipid A is the anchor moiety of LPS in the bacterial membrane and is the active component of LPS responsible for its toxic activity [22]. *Leptospiral* lipid A which differs from *Escherichia coli* (*E. coli*) lipid A stimulating via TLR4 contains unexpected signals through TLR2 [23]. Besides, the cell wall is composed of tightly cross-linked PGN acting like a protective layer for bacteria survival. Cell-wall synthesis and recycling are tightly coordinated to preserve bacterial integrity [24]. Since the survival of bacteria critically depends on their PGN-based cell walls, it is a selective target of many antibiotics. Hence, the development of antibiotic resistance in bacteria, such as *E. coli* and *P. aeruginosa*, is intimately tied with cell-wall synthesis and recycling associated with PGN [25]. In addition, a line of evidence has shown that pretreatment of kidney epithelial cells with outer membrane proteins from pathogenic *Leptospira* triggered the significant expression of tubulointerstitial nephritis-related genes [19,26]. Surface-exposed antigens, due to their location, are likely involved in primary host-pathogen interactions, adhesion, and/or invasion [27]. These host-pathogen interactions are followed by bacterial adhesion to tissues, immune responses, and eventually, bacteria escape of the host immune system [28]. Several *Leptospira* virulence factors that interacted with the host cell immune system components are discussed as follows.

**Figure 1:**
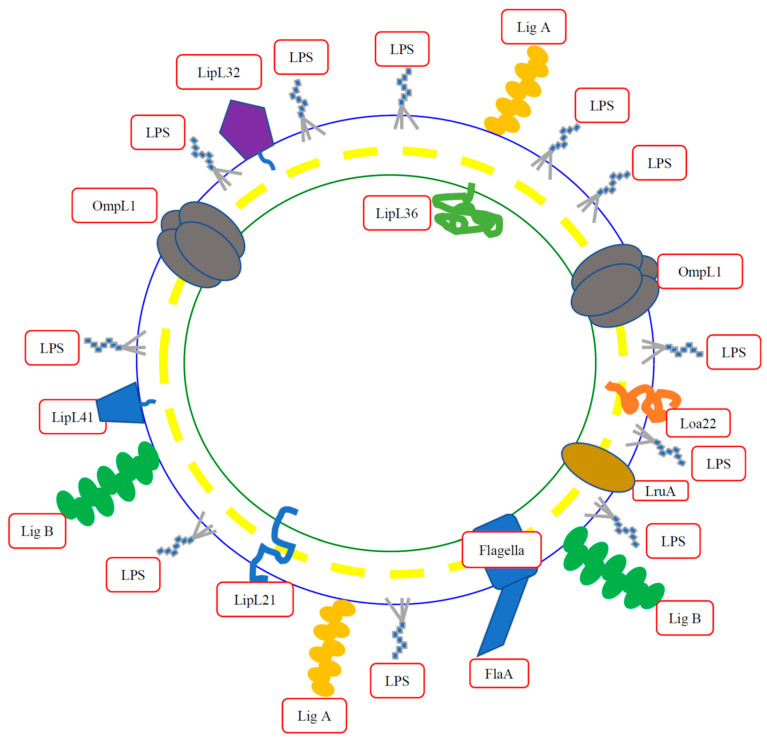
*Leptospira* membrane components. *Leptospria* membrane is a double membrane structure and it is the first line to contact with the host cell and many outer membrane components may act as virulence factors. The virulence factors evaluated by genes manipulation (including LPS, LipL32, Loa22, LipL41, LruA, LigA, LigB, and LipL21) will be selected to discuss in this review for the virulence mechanism investigation. Blue circle, outer membrane; yellow circle, cell wall; green circle, inner membrane.

The most intuitive way to judge whether these possible virulence factors are the main cause of the disease is to use gene knockout/knockdown technology to assess the pathogenicity of the virulence factors. It is currently known to use different gene knockout/knockdown techniques for the evaluation of pathogens in the virulence factors including LPS [29], LipL32 [30], Loa22 [31], LipL41 [32], LipL71 [33], LigA [34], LigB [34,35], and LipL21 [36] (Figure 1). The results of genetic manipulation in pathogenic *Leptospira* demonstrated that some genes are related to the pathogenicity of leptospirosis (including LPS, Loa22, LipL71, and LipL21), while some genes are not related to the pathogenicity of leptospirosis (including LipL32, LipL41, LigA, and LigB). These reports point out the usage of different genetic manipulation methods to knockout or replace specific genes in *Leptospira* to verify its pathogenicity. In Loa22, LipL71, and LipL21, the authors used transposon to obtain *loa22^—^*, *lipl71^—^*, and *lipl21^—^* mutations and evaluated the virulence of the mutation strains in the hamster model. Results demonstrated that Loa22, LipL71, and LipL21 are essential for *Leptospira* virulence [32,34,37]. Surprisingly, the mutation of the major outer membrane lipoprotein, LipL32, showed no significant difference as compared to the WT *Leptospira* in pathogenicity [30]. In LipL41, the authors used transposon to obtain *lipl41^—^* mutation and evaluated the virulence of the mutation strain in the hamster model. Results demonstrated that LipL41 is not essential for *Leptospira* virulence [32]. In LigA and LigB, the authors used transcription activator-like effectors (TALEs) to repress the expression of the LigA and LigB and the results demonstrated that LigA and LigB played potential roles in *Leptospira* pathogenicity [34]. However, another report indicated that using the site-directed homologous recombination method to obtain the *ligb^—^* mutation did not show decreased virulence compared to the WT strain in the hamster model [35]. In this review article, the role of these possible virulence factors at the outer membrane of *Leptospira* will be discussed in the following.

### 2.1. LPS

LPS are large molecules consisting of a lipid and a polysaccharide composed of O-antigen, and LPS are found in the outer membrane of gram-negative bacteria. It is generally believed that TLR4 can recognize the LPS of gram-negative bacteria, while TLR2 is a lipoprotein. However, previous studies have found that the LPS of *Leptospira* is different from that of gram-negative bacteria [23]. Using the characteristics of CHO cells that do not express TLR2 and using TLR2 knockout mice directly confirmed that LPS of gram-negative bacteria can stimulate inflammatory responses, but LPS of *Leptospira* cannot stimulate these cells and animals to produce inflammatory responses. In *Leptospira*, lipid A components of LPS have been shown to have a greater stimulatory inflammatory activity than LPS, perhaps due to the low endotoxin activity of *Leptospiral* LPS [21]. The endotoxic lipid A is the anchor moiety of LPS in the bacterial membrane and possesses the active component of LPS responsible for its toxic activity [22]. Lipid A from *E. coli* activates host pathways via TLR4, but *Leptospira* lipid A, unexpectedly, signals through TLR2 [23]. The recent completion of *Leptospira* genomic sequences has elucidated and identified factors possibly involved in host recognition [37,38]. The first LPS biosynthetic (*rfb*) locus and chemical composition of LPS were identified from *L. interrogans* serovar Copenhageni [39,40]. Genomic data for *L. interrogans* serovars Lai and Copenhageni indicate that synthesis of LPS is encoded in large loci of approximately 100 kb all on one strand of the large chromosome [37,41]. In the *rfb* loci of the *L. santarosai* serovar Shermani genome, there are 64 predicted genes, of which 23 are related to LPS biosynthesis and 11 have been assigned as putative LPS biosynthesis proteins based on functional prediction. Importantly, the *L. santarosai* genome encodes orthologues of *L. interrogans lpx* genes, which are responsible for lipid A assembly, indicating that the enzymatic pathway for lipid A biosynthesis is conserved in *Leptospira* species. The deletion or mutation of LPS in *Leptospira* was performed by using transposon to insert in the LPS biosynthesis genes and two mutation strains, M895 and M1352, were obtained by insertion into LA1641 and rmlC genes, respectively. The two mutation strains altered LPS compositions and further attenuated the pathogenicity of *Leptospira* [29].

### 2.2. LipL32

**Figure 2:**
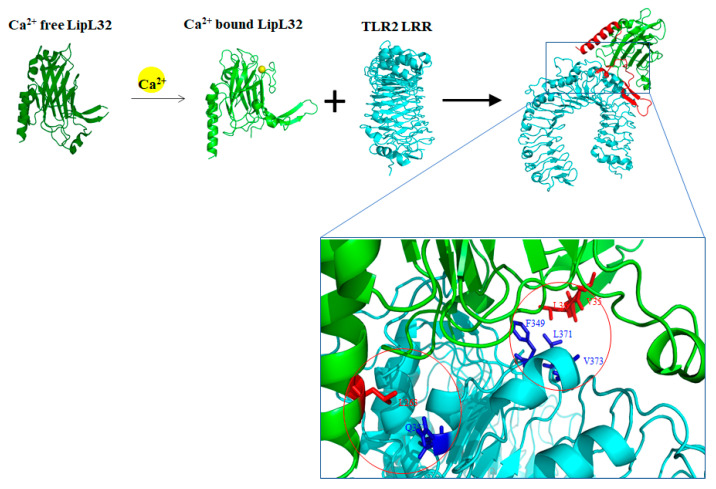
The proposed model of LipL32 and TLR2 interactions. Calcium ion-induced conformational changes of LipL32 and regulated the affinity of LipL32 to TLR2. The Ca^2+^ bound LipL32 interacted with TLR2 and the domains and residues involved in the dimer formation were evaluated. The TLR2 LRR domain is shown in cyan and the LipL32 in green as well as the two termini of LipL32 in red.

Among these *Leptospira* outer membrane components, LipL32 is the most abundant outer membrane component found in the pathogenic *Leptospira*, but not in non-pathogenic ones [2,42]. LipL32, a lipoprotein with lipid modification at its Cys^20^ residues and a signal peptide tag at N terminus [20]. The crystal structure of LipL32 reveals the jellyroll fold structure and demonstrates a calcium ion as an important factor in structural and thermal stability [43,44,45]. In addition, LipL32 has been validated the affinity to the extracellular matrix (ECM) including laminin, collagen I, collagen V, collagen IV, collagen XX, plasminogen, and fibrinogen, while the C terminal and intermediate domain of LipL32 are responsible for the interaction [46,47,48]. Besides, the purified LipL32 protein was capable of increasing the permeability and decreasing the expression of zonula occludens-1 (ZO-1) and inducing the expression of F-actin in human umbilical vein endothelial cells (HUVEC) [49]. Our previous studies indicated that LipL32 induce tubulointerstitial nephritis in mice through mediating pro-inflammatory cytokines gene expression in the proximal tubule cells [50]. Toll-like receptor 2 (TLR2) has been reported to be involved in the LipL32-stimulated chemokine secretion [51]. In *Leptospira*, it has been proven that the LipL32 stimulated inflammatory responses through TLR2 and the calcium-binding cluster (including Asp^132^, Thr^133^, Asp^164^, Asp^165^, and Tyr^178^) of LipL32 that could regulate the affinity of LipL32 and TLR2 upon infection of *Leptospira* [7,42]. We further characterized the interaction between LipL32 and TLR2 protein in several ways to identify the vital domain of LipL32 and the binding mechanism of the LipL32-TLR2 complex. The lipL32-TLR2 complex was predicted from the Cluspro website and the top ten binding models were divided into three types according to the different binding domains. In the three models, the N terminal β1β2 domain, central loop-α3-loop domain, and C terminal α4 helix domain of LipL32 might play a vital role in association with TLR2. Therefore, different truncated LipL32 variants were constructed and characterized. LipL32WT protein was observed to co-localize with TLR2 on HEK293 cell surface as detected by confocal microscopy, while the different co-localization behavior of truncated LipL32 variants was also presented. The corresponding inflammatory responses provoked by different truncated variants of LipL32 were measured by real-time PCR to identify the active domains of LipL32. In addition, the interaction of LipL32 and TLR2 was explored by AFM and ELISA to identify the role essential domains of LipL32 involved in TLR2 interaction. These results indicate that the N terminus of LipL32 might be involved in TLR2 interaction and the C terminus might assist the complex formation (Figure 2). Interestingly, the LipL32 loop-α3-loop domain (LipL32ΔCenα3) might play the regulatory role in the LipL32-TLR2 complex in the presence and absence of Ca^2+^ ion. Further identifying the role of these important domains of LipL32, the essential residues within the three domains of LipL32 probably involved in TLR2 interaction were mutated by site-directed mutagenesis. The single point mutation of L36S, P235S, and L263S variants significantly decreased the affinity between LipL32 and TLR2 while F34S, V35S, F234S, and L263S variants slightly decreased the binding affinity as compared to LipL32WT. Whereas, D195A and D196A variants altered the Ca^2+^ stimulated LipL32-TLR2 complex formation indicated that the two negatively charged residues at loop-α3-loop domain insulated the interaction between LipL32 and TLR2 (Figure 2). Although LipL32 has many functions related to the pathogenicity of *Leptospira*, it was found in the gene knockout experiment that LipL32 knockout *Leptospira* does not affect its pathogenicity [30]. It is speculated that the reason may be because the outer membrane of *Leptospira* contained many functionally similar or functionally complementary genes in *Leptospira* and these genes can maintain the pathogenicity of *Leptospira* when LipL32 is knocked out.

### 2.3. LipL21

LipL21 is the second most abundant protein in the outer membrane of *L. interrogans* serovar Lai. Alignment of the LipL21 sequence from six strains of *Leptospira* revealed 96 to 100% identity [52]. The blast analysis of the *lipl21* gene revealed the presence of *lipl21* in the pathogenic species, but not in non-pathogenic species, indicating that this protein is a virulence factor [53]. LipL21 was found to incorporate with the lipidation at the N-terminal domain and is considered as the lipoprotein [52]. Furthermore, LipL21 was isolated together with other known *Leptospira* OMPs by both Triton X-114 extraction and sucrose density gradient membrane fractionation [54]. The recombinant LipL21 was found to interact with glycosaminoglycans (GAGs), collagen IV, laminin, E-cadherin, and elastin and these interactions of LipL21 to host cell components represented important steps in adhesion, invasion, and evasion of the immune system [55]. Interestingly, many pathogens can use GAG to help their adhesion and invasion. However, LipL21 and OmpL1 are the only two virulence factors to bind GAG in *Leptospira* [55]. In addition to being used as an adhesin, LipL21 has also been found to be a potent inhibitor of neutrophil myeloperoxidase [56]. Inhibition of neutrophil myeloperoxidase can inhibit the neutrophil to produce HOCl, a strong oxidant so that the neutrophil cannot have a toxic effect on *Leptospira* [57]. Besides, LipL21 is demonstrated to bind PGN and the binding of PGN enables *Leptospira* to escape NOD1 and NOD2 recognition. If the PGN of *Leptospira* is not protected by LipL21, PGN will be more easily degraded into muropeptides [36]. The presence of antibodies against LipL21 in patient serum is a reliable marker of *Leptospira* infection and the lipoproteins are an important antigen and play a key role in the pathogenesis of leptospirosis [58] and surface-exposed putative lipoproteins [59,60]. Therefore, LipL21 is a surface-exposed, abundant outer membrane lipoprotein that is expressed during infection and conserved among pathogenic *Leptospira* species. Therefore, the LipL21 interacted with various host cell components for *Leptospira* invasion and bound to PGN molecules to escape NOD1 and NOD2 recognition for immune escape. Interestingly, the LipL21 mutation strain of *Leptospira* showed the increase of PGN digestion by host cells and means *Leptospira* cannot escape detection by NOD1 and NOD2, thereby losing its pathogenicity [36].

### 2.4. Loa22

Loa22 is the first pathogenic factor confirmed in gene knockout experiments and Loa22 knockout mutants have been confirmed in animal experiments to lose the pathogenic ability of *Leptospira* [31]. Loa22 was firstly discovered by the method called PhoA fusion and this protein is present in pathogenic *Leptospira*, but not in non-pathogenic *Leptospira*, indicating that Loa22 protein is probably involved in virulence to host cell [61]. In the genome analysis of a non-pathogenic strain, *L. biflexa* serovar Patoc, it was found that it contains a Loa22-like gene (WP_012390072.1), but the expression of this gene seemed to be downregulated and this protein was not detected in the protein level [62]. Loa22 reacted with convalescent mouse sera and was highly conserved on pathogenic *Leptospira*. Thus, Loa22 could be a candidate for a novel vaccine against *Leptospira* infection [61]. The domain prediction analysis showed that Loa22 protein contained two vital domains including an N-terminal domain (residues 1–77) and an OmpA domain (residues 78–186). According to the prediction of SpLip, Loa22 is a possible lipoprotein with a lipid-modified Cys residue and an atypical Leu residue before this Cys residue or a probable lipoprotein with a cleavage site between residues 20 and 21 to form a mature lipoprotein [63]. From the sequence analysis, Loa22 has sequence homology with other proteins of *L. interrogans* including LA4337, LA3685, LA0056, LA3615, and LB328, and these proteins belong to the OmpA family. Loa22 and these OmpA family proteins share sequence similarities in their C terminal domain, whereas the N-terminal domains are different. The OmpA domain of Loa22 is similar to the OmpA protein of *E. coli*, a major outer membrane protein of *E. coli* [64]. The structure predictions for the Loa22 OmpA domain revealed that this domain is the peptidoglycan (PGN) associating motif [65,66]. Proteins containing the OmpA domain revealed a significant structural proportion of anti-parallel β-sheets that were associated with the outer membrane, especially the PGN molecule [67]. The *E. coli* OmpA N-terminal domain was crystallized and the structure was solved as a β-barrel–structured porin and these domains were inserted into the outer membrane lipid bilayer [68]. However, the N-terminal region of Loa22 has no sequence similarity to OmpA, therefore, this domain was not considered for membrane insertion. Because there is no sequence and structure similarity between Loa22 and other OmpA-like proteins in the N-terminal region, these proteins may be structurally and functionally distinct. The role of Loa22 during pathogenesis remains to be determined and the biological function during infection of *Leptospira* needs further investigation. According to the multifunctional role in bacterial physiology and pathogenesis, the OmpA protein of *E. coli* and other Gram-negative bacteria is believed to play a vital role in adhesion to host cells [64,69] and to induce cytokine production by dendritic cells [69,70]. A previous study in immunofluorescence found that Loa22 is a surface-exposed moiety [71]. In a recent study, recombinant Loa22 was shown to bind in vitro to a limited extent with components of the extracellular matrix (ECM), such as plasma fibronectin and collagen types I and IV [72], suggesting that the surface-exposed domain of Loa22 may act as an adhesin. Furthermore, the lipopeptide moieties of spirochetes are potent mediators of the inflammatory response [27]. Loa22, which has a lipobox sequence and lipid-modified properties as a lipoprotein, could contribute to the innate immunity and may, therefore, induce severe disease manifestations by eliciting the host immunopathogenic responses. In our previous study, Loa22 was demonstrated to trigger inflammation responses on the renal tubular cells [62]. Loa22 contained OmpA-like domain was further proved to interact with PGN of *Leptospira*, and two important amino acid residues, Asp^122^ and Arg^143^, were responsible for PGN binding. Besides, the recombinant Loa22 and its variants in the complex with PGN were incubated with HEK293-TLR2 cells that triggering inflammatory responses, including iL8, MCP-1, and TNF-α. In addition, Loa22-PGN was demonstrated to colocalize with the TLR2 receptors on the HEK293-TLR2 cell surface [62].

### 2.5. LRR20

In the genomic analysis of pathogenic *Leptospira* spp., a protein family with specific leucine-rich repeat (LRR) domains has been identified, however, the functions of these LRR proteins in pathogenic *Leptospira* are still unknown [73]. The LRR domain proteins are a large protein family of more than 6000 proteins available in the sequence database and have been identified as viruses, bacteria, archaea, and eukaryotes [74]. Previous studies revealed that pathogenic *Leptospira* contained numerous LRR genes compared to non-pathogenic species. The length of LRR family proteins are various, they contain from a short 20 residues, such as YopM in *Yersinia* [75], to the longer 28–29-residues repeat of the eukaryotic ribonuclease inhibitor [74] in sequence and their presence in several proteins with diverse functions [76]. In *S. agalactiae*, the gene *lrrg* encoded LRRG protein with novel LPXTG surface anchor was suggested to interact with GBS and CBA/ca mice epithelial cells in vitro and stimulate immunoglobulin G responses to protect against lethal challenge with virulent *S. agalactiae* [77]. Some examples of the LRR proteins, such as internalin proteins in *L. monocytogenes* [78], Yop proteins in *Yersinia pestis* [75], SspH, and SlrP from *Streptococcus* [79,80], and LrrA in *T. denticola* [81,82] are well studied.

**Figure 3:**
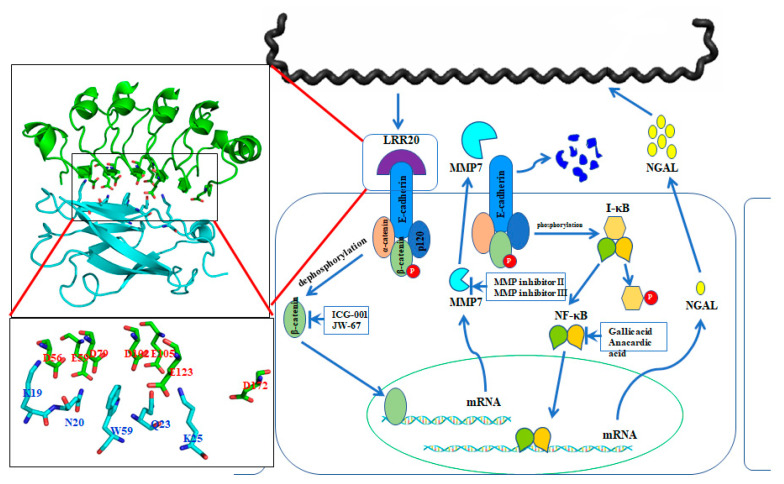
Crosstalk between E-cadherin/β-catenin and NF-κB signaling pathways under *Leptospira* LRR20 treatment. rLRR20 protein interacts with E-cadherin through several vital residues and consequently activates β-catenin. The nuclear translocation of activated β-catenin promotes the expression of its target genes, including MMP7. Subsequently, MMP7 is secreted to the extracellular region. The expression and secretion of MMP7 promote the degradation of E-cadherin on the cell surface and downregulate the cell surface levels of E-cadherin. Meanwhile, the degradation of E-cadherin on the cell surface induces the activation of the NF-κB:p65 signal transduction pathway, which subsequently promotes the expression of downstream target gene NGAL.

The pathogenic bacteria evolved several strategies to invade the host cell [83]. Molecular mimicry is a well-know procedure in the structural mimicry of eukaryotic LRRs by pathogens to compete with the functions of the host to adhere and invade host cell [84]. Most LRR domains are involved in protein-ligand or protein-protein interactions, and proteins containing these domains are mainly found in cell adhesion factors, hormone receptors, and enzyme inhibitors [76,83,85]. Structurally, LRR domains consist of tandems of two or more repeat units forming a curved horseshoe structure [85]. The overall topology of LRR domains depends on the sequence and the number of repeat units [86]. In bacteria, the LRR family protein can further be divided into three subfamilies, including bacterial LRR, SDS22-like LRR, and Tp-LRR [74,80]. Specifically, the LRR proteins with the TpLRR family are considered to associate with bacterial cell surfaces, and the following components were identified as TpLRR family, including TpLRR protein from *T. pallidum* [87], BspA from *T. forsythensis* [88], and PcpA from *S. pneumoniae* [89]. In this study, we focus on *L. santarosai* serovar Shermani and the characterization of the LRR proteins for their structural and functional studies. There are thirteen LRR genes from pathogenic *L. santarosai* serovar Shermani including LSS_00195, LSS_00880, LSS_00914, LSS_00919, LSS_01692, LSS_01912, LSS_02172, LSS_07304, LSS_11580, LSS_15741, LSS_16811, LSS_17860, and LSS_18324 [38,90]. Among these LRR domain proteins, LSS_11580 (LRR20) protein was the first LRR domain protein in *L. santarosai* for structural and functional studies. The crystal structure of LRR20 from pathogenic *L. santarosai* was solved by X-ray crystallography and the function of LRR20 was found to interact with human E-cadherin (Figure 3). As compared to the known structure LRR proteins from *L. interrogans*, LRR20 shows a relatively low identity to the four LRR proteins including LIC12234, LIC10831, LIC11098, and LIC12759 [83]. Besides, the crystal structure of LRR20 was solved by X-ray diffraction at 1.99 Å resolution that contains seven α-helices and five β-sheets in the 3D structure [91]. rLRR20 was demonstrated as an E-cadherin binding protein and interacted with the EC1 domain of E-cadherin through charged-charged interaction. Three vital residues (D56A, E59A, and E123A) were proposed to involve in the interaction between rLRR20 and EC1 domain of E-cadherin [91]. Cytokine array study also demonstrated that the rLRR20 induced neutrophil gelatinase-associated lipocalin (NGAL) expression in kidney epithelial cells [91]. Further investigation of the role of rLRR20 in leptospirosis revealed rLRR20 was observed to colocalize with E-cadherin on the cell surface and activate the downstream transcription factor, beta-catenin, which subsequently promoted the expression of MMP7, a kidney injury biomarker. To confirm the signal transduction pathway, MMP7 inhibitors were used to demonstrate that the secreted MMP7 degrades surface E-cadherin. This feedback inhibition mechanism downregulated surface E-cadherin expression and inhibited the colonization of *Leptospira*. The degradation of surface E-cadherin was also found to activate the NF-kB signal transduction pathway. Leptospirosis-associated acute kidney injury is associated with the secretion of NGAL, a downstream upregulated biomarker of the NF-kB signal transduction pathway. The crosstalk between E-cadherin/β-catenin and NF-kB signal transduction pathways during *Leptospira* infection was, therefore, proposed in Figure 3 [92]. Thus, rLRR20 of *Leptospira* induces kidney injury in host cells and inhibits the adhesion and invasion of *Leptospira* through the upregulation of MMP7 and NGAL.

### 2.6. LipL71

LipL71 is a lipoprotein with a molecular mass of 71 kDa in *Leptospira* and the protein was found to modify the palmitate acid for the lipidation. The lipid modification enables LipL71 anchored to the inner membrane and outer membrane of *Leptospira* [93]. LipL71 is also called LruA due to the first discovered protein in the *Leptospira* recurrent uveitis and the protein was not observed in non-pathogenic *Leptospira* indicating that the protein was involved in *Leptospira* pathogenesis [93]. The functional domain analysis indicated that LipL71 contained the LysM domain and this domain was responsible for PGN binding [33]. Interestingly, when the horse is infected with *Leptospira* and the LipL71 protein is expressed in the eyes of uveitic horses at a high level, the eye will induce specific antibodies against the *Leptospira* protein, especially LipL71. The antibody against LipL71 was also found to cross-react with alpha-crystallin B, beta-crystallin B2, and vimentin in the eye fluid, and the cross-react of the antibody contributed to the severity of this eye disease [94]. In the transposon mutation analysis, LipL71 was demonstrated as the virulence factor and required for the *Leptospira* pathogenesis [33]. In the animal model, the LipL71 mutation strain was inoculated to hamsters and results showed the survival of all hamster models indicating that the LipL71 mutation strain is non-pathogenic [33]. This study also suggested that proteins involved in PGN binding play an important role in the pathogenicity of *Leptospira*.

### 2.7. LigA and LigB

The *Leptospira* contains a class of high molecular weight immunoglobulin-like repeat antigen molecules, called the Lig protein family [95]. The Lig family contains three members, namely LigA, LigB, and LigC, which belong to bacterial immunoglobulin-like (Big) repeat members in classification [95]. The main function of proteins with this type of domain is related to the role of host-pathogen and previous reports indicated that the recombinant *Leptospira* Lig domain protein interacted with the ECM, such as fibronectin, fibrinogen, collagen, and laminin [96,97]. Therefore, the Lig domain proteins were considered as the virulence factors for leptospirosis. However, the genetic manipulation of the *Leptospira* knockout in LigB protein using the homologous recombination method indicated that the *ligB* mutant showed no reduction in virulence compared to the wild-type strain in a hamster model of leptospirosis. Besides, inoculation of rats with ligB mutants induced persistent colonization of the kidneys. Finally, LigB is not required to mediate bacterial adhesion to cultured cells [35]. In contrast, other groups also showed the knockout experiments to obtain the LigA and LigB mutation strains using the TALE method and the results indicated that the decreased levels of LigA and LigB protein expression result in decreased virulence in hamsters, which may indicate cumulative roles of LigA and LigB in pathogenesis [34].

### 2.8. LipL41

LipL41 is first found at the outer membrane of *Leptospira*, and LipL41 is the third most abundant protein in the outer membrane of *Leptospira* [27]. LipL41 uses Cys residue to modify lipid molecules to form lipoproteins that anchor at the outer membrane of *Leptospira*. LipL41 is only expressed in pathogenic *Leptospira*, not in non-pathogenic *Leptospira*, therefore, LipL41 is also considered to be an important virulence factor. The amino acid sequence analysis indicated that LipL41 is highly conserved in the different serotypes of pathogenic *Leptospira*, so some studies have also used LipL41 as an antigen used as a serodiagnostic target. However, the function of LipL41 in *Leptospira* is still unclear. It is pointed out in the literature that LipL41 does not induce an inflammatory response [98]. In the gene knockout experiment, LipL41 is not required and is also the main factor that causes hamster disease [32]. In addition, LipL41 has been considered as a heme-binding protein, and the binding mechanism of LipL41 and heme is speculated to be related to the heme-binding pocket formed by Cys^40^-Ser and Cys^220^-Pro in the composition of LipL41 protein [98]. LipL41 was found to co-express with another chaperon protein, Lep, which helps LipL41 express and fold into a 36-mer bilayer protein particle structure [55,98].

## 3. The Diagnosis of *Leptospira*

The diagnosis of *Leptospira* is still an important topic in the clinical scenario because leptospirosis is often misdiagnosed with other febrile diseases at the beginning of leptospirosis. Clinical diagnosis of *Leptospira* infection is difficult as the symptoms are similar to various bacteria and viruses infections, such as malaria, dengue fever, rickettsial diseases, yellow fever, and HIV infections; therefore, laboratory support for confirmation is indispensable [99]. Therefore, a clear diagnosis of leptospirosis depends entirely on laboratory confirmation.

### 3.1. Serological Diagnosis of Leptospirosis

Among all currently available diagnosis methods, the serum microscope agglutination test (MAT) is still the gold standard for leptospirosis. However, MAT diagnosis of *Leptospira* is not widely used due to several limitations: (i) the preparation of the culture medium is difficult; (ii) the maintenance of the live *Leptospira* strains is difficult; (iii) different serovars of *Leptospira* cultures are easily mixed up and specific antibodies should be used to identify the diagnostic strains regularly; (iv) *Leptospira* culture mediums are easily contaminated by nonpathogenic *Leptospires* and other bacteria; (v) MAT can be used for the diagnosis of leptospirosis only when the *Leptospira* serogroups are known [100]; (vi) MAT is inappropriate when there is a lack of prior knowledge of epidemic pathogenic strains in an environment where resources are scarce and laboratory facilities and skilled laboratory personnel are limited [101]; (vii) MAT may also delay the identification and treatment of the disease because IgG and IgM only appear from day 5 to day 7 after *Leptospira* infection [102]. According to the reasons mentioned above, other diagnostic methods are, therefore, developed for *Leptospira* diagnosis including ELISA, indirect fluorescent antibody test (IFAT), macroscopic slide agglutination test (MSAT), latex agglutination tests, such as the DriDot, and various lateral flow assays.

### 3.2. Bacteria and Molecules Diagnosis of Leptospirosis

Besides the serovar testing, diagnosis of *Leptospira* by culturing and isolating cells from clinical samples is beneficial because the cells can be confirmed by their specific morphology. However, the growth of *Leptospira* may take a long time (from weeks to months) and the long period of cell culture may also cause delays in the diagnosis and treatment of the patients. Moreover, the sensitivity of the cell culture method was estimated to be less than 23% [103]. In addition, quantitative PCR provided the most reliable diagnosis result of *Leptospira* and qPCR showed high reliability, sensitivity, and specificity. Many previous studies have developed and validated qPCR tests for specific genes, such as the *rrs* gene [104], *lipL32* gene [105], and *lfb1* [106]. However, there are still many problems to be overcome when using qPCR to detect *Leptospira* including the need for stable DNA extraction technology, technical expertise, and expensive instruments are required. For the use of qPCR for *Leptospira* screening, a holistic system is necessary to ensure speed, simplicity, and cost-effectiveness. Therefore, a fast and effective detection method is needed for *Leptospira* diagnosis and the use of specific antibodies to detect *Leptospira* antigens in clinical samples can be considered a reliable technique for the diagnosis of leptospirosis. Therefore, many studies are using the pathogenic factors of leptospirosis as the target to develop its antibodies for the diagnosis and treatment of leptospirosis [102]. The pathogenesis of leptospirosis depends on blood dissemination, so *Leptospira* cells can be detected in the patient’s blood and many internal organs. In addition, in the second week of infection, intact *Leptospira* cells can also be found in the urine. Previous studies have demonstrated the usefulness of detecting *Leptospira* cells in urine using monoclonal antibodies [107,108]. A study demonstrated the potential of mAbs for diagnostic applications by immunizing mice to produce mAbs against outer membrane lipoproteins [109].

## 4. Conclusions

Leptospirosis is a neglected zoonotic infectious disease common in tropical and subtropical regions and is often misdiagnosed because its symptoms are similar to many other infectious diseases. The organs that *Leptospira* are prone to are mainly the lungs, liver, and kidneys, among which the kidney is the main colonization organ. At present, many virulence factors of *Leptospira* have been reported. It is confirmed that not all virulence factors have pathogenicity, the main reason may be because there are many virulence factors with similar functions in *Leptospira* that can complement its functions. At present, loss of pathogenicity in *Leptospira* outer membrane components mutation strains (including LPS, Loa22, LipL71, and LipL21) were obtained by using the transposon method and further confirmed the pathogenicity of these virulence factors. Some of these virulence factors seem to have the ability to bind to PGN, therefore, it is speculated that PGN plays a role in the pathogenicity of *Leptospira* and the PGN binding also plays an important role in evading immunity. Analysis of the structure and function of these virulence factors can help to further understand the pathogenic mechanism of leptospirosis and to interpret the interaction between pathogenic *Leptospira* and host cells, which provides important information for future research on leptospirosis. These virulence factors will be used for subsequent targets against *Leptospira* and vaccines to treat and prevent leptospirosis. Finally, these virulence factors will also be used as the antigen to develop the diagnostic tools for early leptospirosis detection and confirmation.

## Data Availability

Not applicable.
